# Nutritional outcomes in pediatric liver transplantation: a longitudinal analysis of anthropometric measures

**DOI:** 10.1590/1984-0462/2025/43/2024273

**Published:** 2025-12-01

**Authors:** Guilherme Baptistello, Thais Oliveira de Sousa, Melina Melere, Caroline Beskow, Cristina Targa Ferreira, Carolina Soares da Silva, Luiza Nader, Flávia Heinz Feier, Antônio Nocchi Kalil, Simone Farías-Antúnez

**Affiliations:** a Universidade Federal de Santa Catarina, Araranguá, SC, Brazil.; b Hospital da Criança Santo Antônio, Porto Alegre, RS, Brazil.; c Centro Universitário Metodista IPA, Porto Alegre, RS, Brazil.; d Irmandade Santa Casa de Misericórdia de Porto Alegre, Porto Alegre, RS, Brazil.

**Keywords:** Liver transplantation, Pediatric, Anthropometry, Nutritional status, Transplante de fígado, Criança, Antropometria, Estado nutricional

## Abstract

**Objective:**

To evaluate the nutritional outcomes in patients aged zero to 18 undergoing liver transplantation, comparing preoperative and postoperative periods, and to determine the average time required for malnourished patients to reach eutrophy.

**Methods:**

This retrospective longitudinal study utilized a non-probabilistic convenience sample of 66 children and adolescents who underwent liver transplantation at a hospital in a Brazilian capital. Anthropometric data (weight, height, and body mass index (BMI)-for-age) were extracted from medical records and expressed as Z-scores according to the World Health Organization (WHO) growth charts for sex and age. Statistical analyses were performed using the paired Student’s t-test and the Kaplan-Meier survival curve.

**Results:**

Before the transplant, 43.94% of the patients were malnourished. Following the procedure, significant increases were observed in mean weight (from 12.47 kg to 16.63 kg; p<0.0001), height (from 80.09 cm to 91.51 cm; p<0.0001), and BMI (from 16.60 kg/m^2^ to 18.29 kg/m^2^; p<0.0001). The Z-scores for weight-for-age (-1.16 to 0.23; p<0.0001), height-for-age (-1.55 to -1.28; p=0.024), and BMI-for-age (-0.29 to 1.61; p<0.0001) also improved. The average time to reach eutrophy was 228 days.

**Conclusions:**

Liver transplantation significantly improved the nutritional outcomes of children and adolescents, as evidenced by improvements in anthropometric parameters. The mean time to reach eutrophy was approximately 7.5 months.

## RESUMO

**Objetivo:**

Avaliar a evolução nutricional de pacientes entre zero e 18 anos submetidos a transplante hepático, comparando os períodos pré e pós-operatório, e determinar o tempo médio para que pacientes desnutridos atinjam a eutrofia.

**Métodos:**

Estudo longitudinal retrospectivo com 66 crianças e adolescentes que realizaram transplante hepático em um hospital de uma capital do Brasil. Dados antropométricos (peso, altura e índice de massa corporal — IMC) foram coletados e expressos em escores Z. Análise com teste *t* de Student para amostras pareadas e curva de Kaplan-Meier.

**Resultados:**

No pré-transplante, 43,94% estavam desnutridos. Após o transplante, houve aumento significativo nas médias de peso (12,47 kg para 16,63 kg; p<0,0001), estatura (80,09 cm para 91,51 cm; p<0,0001) e IMC (16,60 kg/m^2^ para 18,29 kg/m^2^; p<0,0001). Os escores Z de peso para idade (-1,16 para 0,23; p<0,0001), estatura para idade (-1,55 para -1,28; p=0,024) e IMC para idade (-0,29 para 1,61; p<0,0001) também melhoraram. O tempo médio para atingir a eutrofia foi de 228 dias.

**Conclusões:**

O transplante hepático melhorou o estado nutricional das crianças e adolescentes, refletido pelo aumento nos parâmetros antropométricos. Verificou-se que o tempo médio para atingir a eutrofia foi aproximadamente 7,5 meses.

## INTRODUCTION

The liver is essential for managing energy and nutrient metabolism. Liver diseases lead to complex pathophysiological disturbances affecting nutrient digestion, absorption, distribution, storage, and utilization. In pediatric patients, chronic liver diseases are predominantly characterized by cholestatic.^1^ Research indicates that malnutrition affects between 65 and 100% of patients with chronic liver diseases.^2^

Malnutrition, a multifactorial condition prevalent in children with liver disease, significantly increases the risks of morbidity and mortality. Therefore, maintaining an adequate nutritional status in children with chronic liver disease can prevent severe hepatic complications.^3^

Moukarzel et al. research established a strong correlation between nutritional status and liver transplant outcomes in pediatric patients. The findings suggest that children with poor nutrition are more prone to infections after transplant, surgical complications, and a higher chance of mortality.^4^ In addition, malnutrition in kids with end-stage liver disease can lead to long-term challenges after the transplant, including delayed cognitive development and slower growth.^1,3^

Thus, it is evident that malnutrition is a critical modifiable factor both before and after liver transplantation.^3^ For this reason, assessing nutritional status is crucial, with anthropometric evaluation being highlighted as a simple, convenient, cost-effective, efficient, quick, and non-invasive approach.^5^ Longitudinal assessment of nutritional status, primarily using height-for-age, but also weight-for-age and body mass index (BMI)-for-age indicators, has proven to be an effective method for monitoring the nutritional condition of liver transplant patients over the long term and is already routinely collected in clinical practice. These measures provide a precise and comprehensive evaluation of nutritional status, aiding in more accurate clinical management.^6^

Liver transplantation often becomes a child’s only chance of survival in case of severe liver pathology, and the consideration of the nutritional aspect is an integral component of its success. Liver transplantation represents a vital intervention for children with advanced liver disease, where comprehensive nutritional assessment is key to supporting successful outcomes. This study contributes meaningfully by analyzing changes in anthropometric measures and nutritional status among these patients, while also providing an average timeline for malnourished individuals to reach a healthy nutritional state. The findings offer valuable insights to enhance post-transplant care and improve our understanding of the nutritional and anthropometric adjustments experienced by this patient group. The objective of this study was to follow pediatric patients undergoing liver transplantation at the Pediatric Liver Transplantation Service of Hospital Criança Santo Antônio, Santa Casa da Misericórdia, Porto Alegre, Brazil, and to evaluate the impact of nutritional status evolution using anthropometric measures (weight, height, and BMI) in children and adolescents aged zero to 18 years, before and after transplantation.

## METHOD

This study was a retrospective longitudinal analysis. The study included patients aged zero to 18 years treated at the hepatology outpatient clinic of Hospital Criança Santo Antônio, Santa Casa da Misericórdia, Porto Alegre, Brazil, who underwent liver transplantation between 2013 and 2024. Participants were selected according to their liver transplant diagnosis, categorized under the International Classification of Diseases (ICD) code Z94.4. This was a non-probabilistic convenience sample, including all children who underwent liver transplantation at the hospital, with parental authorization obtained through signed Informed Consent Forms. This project was approved by the Ethics Committee of the Irmandade da Santa Casa de Misericórdia de Porto Alegre (ISCMPA) under Certificate of Presentation for Ethical Appreciation — CAEE number 19926219.4.0000.5683 and approval number 3.900.764.

Children and adolescents (ages 0–18) who underwent liver transplantation at the hospital between 2013 and 2024, with parental or legal guardian consent for participation, were eligible to be enrolled in the study. Participants who lacked anthropometric data for the pre- or post-transplant periods, with an average interval of one year, or those who had deceased, were excluded.

The primary outcome was the nutritional status evolution, that was assessed through anthropometric measurements in children and adolescents aged zero to 18 years. The status was determined using weight, height, and BMI measurements, applying weight-for-age, height-for-age, and BMI-for-age indices. Weight was measured as follows: children under two years were weighed either naked or wearing a clean, dry diaper, using a pediatric scale. A P15 Welmy 15 kg digital pediatric scale was used. Children aged two years or older were weighed individually, in a private setting, barefoot, wearing light clothing, and standing unassisted in the center of the electronic scale platform. The Welmy W200A electronic scale was used. All measurements were recorded in kilograms. Height was measured as follows: children under three years had their height measured using an aluminum and plastic infantometer with 1 mm precision. For children aged three years or older, height was measured using a stadiometer equipped with a movable block, also made of aluminum and plastic, with 1 mm precision. This measurement was recorded in centimeters. BMI was calculated using the formula BMI=weight/(height)^2^ and recorded in kg/m^2^. The measurements were taken by a trained data collector, strictly following the techniques and guidelines established by the World Health Organization (WHO). These measurements were standardized according to the WHO growth curves by sex and age and expressed as Z-scores, using the WHO Anthro software (version 3.1) /2010 and WHO Anthro Plus/2007. The Z-score cutoff of -2 was used to classify the nutritional status of children in relation to underweight, stunting, and malnutrition. Children with Z-scores between -1 and -2 were classified as at risk of malnutrition, while those with Z-scores between -1 and +1 were considered eutrophic. Children with Z-scores between +1 and +2 were classified as overweight, and those with Z-scores above +2 were categorized as obese. The change in nutritional status was assessed by comparing the difference in pre-transplant anthropometric parameters with the same parameters after an average of 12 months post-transplant. Pre-transplant anthropometric measurements were collected on the day of liver transplantation. Post-transplant measurements used for comparative analysis were obtained at a median of 356 days (interquartile range — IQR: 343–378) after the transplant. Among malnourished patients, the median time to reach eutrophic status was 124 days (IQR: 79–301). The average time to reach eutrophy was calculated as the number of days between the transplant date and the date when eutrophic status was reached. For children under five years, weight-for-age and/or height-for-age were used to assess nutritional status, while, for children over five years, BMI-for-age, weight-for-age (up to ten years), and/or height-for-age were used. Reaching eutrophy was defined as the normalization of initially inadequate anthropometric parameters. For cases with two or more inadequate parameters, eutrophy was achieved through the correction of at least one of them. For individuals with only one inadequate parameter, eutrophy was achieved by normalizing that specific parameter. Malnutrition was defined as the presence of at least one inadequate anthropometric parameter as mentioned above.^7^

In this study, the independent variables considered were sex (male and female) and age (in years and months). Both sex and age were obtained from the information available in the medical records. The age at which eutrophic status was reached was calculated as the difference between the date of birth and the date when eutrophy was reached.

Data were collected from hospital medical records over the research period. All data from 2013 to May 2024 were extracted from the medical records. Data extraction was performed by two researchers to ensure accuracy through subsequent comparison. The data were meticulously recorded and organized in an Excel spreadsheet for analysis.

Data analysis was performed using Minitab Statistical Software, version 21.4.2. The data were described statistically in terms of mean±standard deviation (SD), median, minimum, and maximum (for quantitative variables), and percentages (for qualitative variables), where applicable. The analysis included a paired Student’s t-test to assess statistically significant differences between the means of the variables before and approximately 12 months after transplantation. Additionally, the average time for malnourished patients to reach eutrophic status was calculated using a Kaplan-Meier curve. The level of significance was set at p<0.05.

## RESULTS

The sample studied was made up of 66 children and adolescents who had undergone liver transplantation, of whom 54.55% were male and 45.45% were female. The average age at transplantation was 2.59 years, with a standard deviation of 3.7 years. Of the patients, 29 were malnourished before the transplant, representing 43.94% of the total. Table 1 presents the basic demographic data.

**Table 1:** Population characteristics.

Basic study characteristics	n	%	Mean±SD
Total patients	66	100	
Eutrophic patients (pre-transplant)	37	56.06	
Malnourished patients (pre-transplant)	29	43.94	
Sex			
Male	36	54.55	
Female	30	45.45	
Age at transplant			2.59±3.70 (years)

Regarding the characteristics of the anthropometric variables of interest in the pre- and post-transplant periods, the weight showed an increase from the pre-transplant period to the post-transplant period, with a mean difference of 4.16 kg (t=-10.06, p<0.0001). Height also exhibited a positive change, with an average increase of 11.42 cm (t=-17.72, p<0.0001). BMI increased by 1.69 kg/m^2^ after the transplant (t=-5.56, p<0.0001). The weight-for-age Z-score improved by 1.39 (t=-10.08, p<0.0001), while the height-for-age Z-score showed a more modest increase of 0.27 (t=-2.31, p=0.024). Lastly, the BMI-for-age Z-score saw a substantial improvement of 1.90 (t=-6.58, p<0.0001). Table 2 summarizes the pre- and post-transplant anthropometric data.

**Table 2:** Comparison of pre- and post-transplant anthropometric measurements and Z-scores.

Variables	Mean±SD	Median	Minimum	Maximum
Pre-transplant				
Weight (kg)	12.47±11.88	7.42	4.30	64.70
Height (cm)	80.09±25.85	68.50	56.00	161.00
BMI	16.60±2.67	16.24	11.31	25.10
Weight-for-age Z-score	-1.16±1.45	-1.09	-3.00	2.29
Height-for-age Z-score	-1.55±1.28	-1.63	-3.00	2.48
BMI-for-age Z-score	-0.29±1.72	-0.39	-4.53	4.12
Post-transplant				
Weight (kg)	16.63±11.97	12.18	7.34	78.00
Height (cm)	91.51±22.20	83.00	71.50	165.00
BMI	18.29±2.71	18.15	14.20	33.76
Weight-for-age Z-score	0.23±1.21	0.22	-2.45	2.77
Height-for-age Z-score	-1.28±1.23	-1.20	-3.65	0.60
BMI-for-age Z-score	1.61±2.31	1.49	-1.41	17.30

SD: standard deviation; BMI: body mass index.

All pre- and post-transplant variations in weight, height, BMI and their respective Z-scores showed statistically significant differences (p<0.05). Pre-transplant anthropometric measurements were collected on the day of liver transplantation. Post-transplant measurements used for comparative analysis were obtained at a median of 356 days (IQR: 343–378) after the transplant. Among malnourished patients, the median time to reach eutrophic status was 124 days (IQR: 79–301).

**Figure 1:**
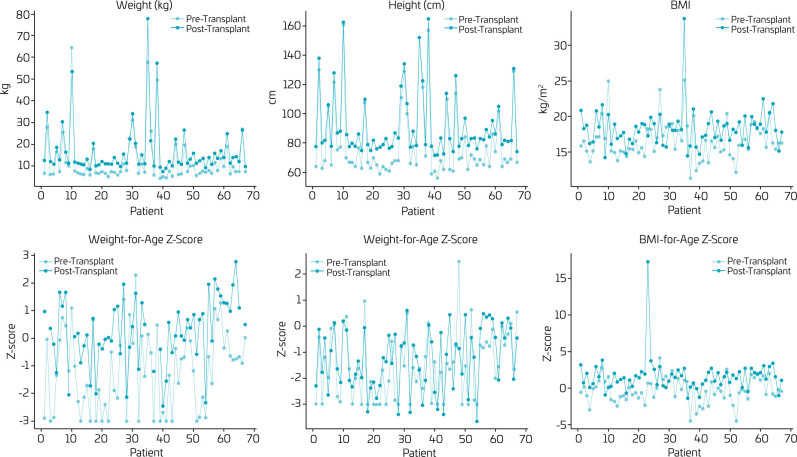
Variations in anthropometric measurements (weight, height, and BMI) and their Z-scores in children undergoing liver transplantation: a comparison before and after the procedure.

Figure 1 presents a set of graphs comparing anthropometric variables (weight, height, and BMI) and Z-scores (weight-for-age, height-for-age, and BMI-for-age) for each patient before and after liver transplantation, with an average follow-up interval of 12 months.

BMI: body mass index.

**Figure 2:**
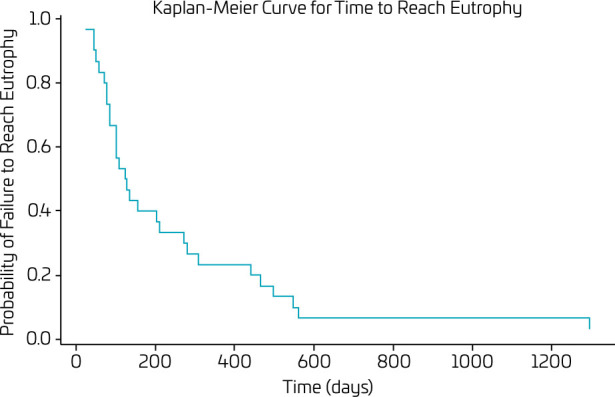
Survival analysis: Kaplan-Meier curve for time to reach eutrophy.

Figure 2 presents the Kaplan-Meier curve used to evaluate the time required to reach eutrophy in patients who were malnourished before liver transplantation. The analysis was based on data from 29 patients who were initially malnourished in the pre-transplant period, considering the time in days from the transplant to the moment they reached eutrophy. The Kaplan-Meier curve illustrates the probability of not reaching eutrophy at various points after transplantation. Each step in the curve represents a patient reaching eutrophy, thereby reducing the overall probability of not achieving this status.

The average time to reach eutrophy was 228 days (SD±261). The first quartile was 77 days, indicating that 25% of patients reached eutrophy within this time or less. The median time was 123 days, indicating that half of the patients reached eutrophy within this period. The third quartile was 280 days, indicating that 75% of patients reached eutrophy within this time frame. Finally, the minimum time recorded to reach eutrophy was 26 days, while the maximum was 1,294 days.

## DISCUSSION

Anthropometric measurements are regarded as the most practical and objective parameters for assessing the nutritional status of children undergoing liver transplantation.^6^ A significant proportion of the children and adolescents undergoing liver transplantation exhibited nutritional deficiencies during the preoperative period. Conventional anthropometric measurements, including weight-for-age, BMI-for-age, and height-for-age, revealed malnutrition in approximately 43.94% of the cases. This finding is consistent with the study by Ferreira et al., which reported similar percentages of pediatric patients with chronic liver disease.^2^ The pathophysiology of this malnutrition is intrinsically linked to liver dysfunction, which alters the metabolism of carbohydrates, lipids, proteins, and vitamins, while impairing intestinal nutrient absorption.^8^ Additionally, children with chronic liver disease exhibit a hypermetabolic state, characterized by increased energy expenditure.^9^ Inflammatory activity, such as elevated interleukin-6 levels, also induces the loss of lean body mass, contributing to nutritional imbalance.^10^ In this context, it is evident that patients with chronic liver disease (CLD) require increased energy intake, which can reach up to 150% of the predicted value for their height and weight.^9^ If this intake is not met, gradual deficiencies in weight, height, and BMI occur.

In this study, the average age at transplantation was 2.59 years, primarily due to the leading indication for transplantation: bile duct obstruction, particularly cholestatic biliary atresia. This finding aligns with current literature, as guidelines from the American Association for the Study of Liver Diseases (AASLD), the American Society of Transplantation (AST), and the North American Society for Pediatric Gastroenterology, Hepatology, and Nutrition (NASPGHAN) indicate that most patients with biliary atresia undergo liver transplantation before the age of four.^11^

This study found significant improvements in the nutritional status of patients, with the intervention leading to positive changes in all anthropometric parameters (weight, height, and BMI), as well as their corresponding Z-scores. Both initially malnourished and eutrophic patients demonstrated notable progress, underscoring the positive and significant impact of liver transplantation on the health of these children. This is largely because liver transplantation corrects the underlying metabolic and nutritional deficiencies associated with end-stage liver disease. This improves the absorption and utilization of nutrients, facilitating weight gain and growth. This recovery is evidenced by improvements in weight, BMI, and their respective Z-scores during the post-transplant period. This is consistent with the normalization of the insulin-like growth factor (IGF) axis post-transplantation-a factor that is very important in aiding growth and weight gain.^12^ Another relevant finding is that the statistical analysis corroborated the studies by Hammad et al., demonstrating that the greatest relative weight gain occurs within the first six months post-operation and that full recovery of weight is generally achieved within the first year after transplantation, especially in patients with depleted total body mass.^13^

In this study, height exhibited the least variation among the anthropometric parameters between pre- and post-transplant Z-scores. This is because children under 24 months at the time of transplantation exhibited significant growth during the first year after transplantation, reaching height distributions comparable to those of age-matched populations. Although older children showed growth improvements, they often remained stunted compared to their peers.^14^ This is because older children have less time to catch up in growth before puberty, when most linear growth occurs.^15^ Another factor contributing to reduced height gain, as observed by Jara et al., is the use of daily steroid therapy.^16^ In light of this, a systematic review and meta-analysis examined the relationship between corticosteroid use after solid organ transplantation, finding that drug withdrawal was associated with significant growth improvements compared to continued steroid use.^17^ However, the practice of corticosteroid withdrawal remains controversial, primarily due to the risk of rejection and potential long-term graft loss associated with its absence.^18^ Furthermore, a study by Leiskau et al. found that continuous low-dose steroid therapy was associated with impaired growth two years after transplantation, but not at five years, suggesting that the negative impact of steroids on growth may diminish over time.^19^

In this study, the average time for initially malnourished pediatric patients to reach eutrophy after transplantation was approximately 7.5 months, with a minimum of 26 days and a maximum of three years and six months. A 1997 study reported an average time of 12 months to reach eutrophy after liver transplantation.^20^ This discrepancy can be attributed to differences in sampling methods between studies, as well as variations in the types of analysis and nutritional therapies available at the time. Additionally, recovery times can vary among patients depending on preoperative nutritional status, persistent medical conditions, glucocorticoid use, recurrence of liver dysfunction, chronic rejection, and feeding-related behavioral challenges.^21^ In this analysis, the main complications significantly impacting nutritional status in the short, medium, and long term were of biliary and infectious origin. Biliary complications, including bile leakage and anastomotic strictures, align with data reported in the literature, where these complications occur in approximately 10% of children in the immediate postoperative period and 20% in the long term, making them among the most frequent complications.^22^ Infections also significantly contributed to nutritional deficits due to immunosuppression, as patients became more susceptible to infections, further compromising their nutritional status. A study from Brazil revealed that severe infections affect up to 52% of children who undergo transplants, becoming a major cause of illness, death, and nutritional deficits. These infections can lead to anorexia, nutrient malabsorption, and increased metabolic demands, further exacerbating malnutrition.^1^

Despite its relevant findings, this study has certain limitations. First, the sample was limited to a single medical center, which may not fully represent the pediatric population undergoing liver transplantation in other regions or institutions. However, Hospital da Criança Santo Antônio in Porto Alegre is a reference center for pediatric liver transplantation in Brazil, receiving patients from multiple regions across the country. Furthermore, the research adopted a convenience sampling method in which participants were sampled based on availability rather than randomly. However, all pediatric patients who underwent transplantation during the study timeframe and fulfilled the inclusion criteria were incorporated. Though anthropometric measure was basically the focus of analysis, excluding other relevant nutritional status indicators such as biochemical indicators and general diet, studies have however shown that anthropometric measures are simple, inexpensive, rapid and non-invasive techniques, hence can be used as an effective tool in the assessment of nutritional status.^23,24^ Finally, postoperative complications and variability in the clinical and nutritional management of patients were not fully explored, which may influence the observed nutritional recovery.

The study also highlights positive aspects, offering significant contributions to the literature on the nutritional status of children undergoing liver transplantation. Notably, despite the limitations, the study demonstrated significant improvements in anthropometric parameters, reflecting nutritional recovery after transplantation. This is also a very innovative study regarding the average time spent to reach eutrophy by undernourished pediatric patients who underwent liver transplantation in Brazil. It gives the mean time to eutrophy, which provides nutritional planning for effective post-transplant interventions that help improve the care and health outcomes of the patients.

As the studies show, liver transplant massively improves the nutritional status in children and adolescents with serious liver diseases. Anthropometric data, reviewed pre- and post-transplantation, showed that weight, height, and BMI had significantly improved to further establish the efficacy of liver transplantation in correcting nutritional defects associated with end-stage liver disease. The improvements in the Z-scores for weight, height, and BMI can be interpreted as the fact that liver transplantation not only triggers the process of liver function restoration but also encourages the further growth and considerable nutrition recovery. One of the major and innovative results of the given study, however, is the fact that the average number of days that the pediatric patients took to make it to the eutrophic state was 228 days in the case of children and adolescents who were initially suffering from malnourishment. This finding establishes a temporal reference for post-transplant nutritional recovery and provides a solid foundation for planning more effective and personalized nutritional interventions, thereby optimizing strategies and promoting faster, healthier recovery. These findings can be used as the ground for further multicenter studies to confirm and extend the present observations, thus guiding more efficient clinical practice to optimally improve liver transplant outcomes in pediatric patients.

## Data Availability

The database that originated the article is available with the corresponding author.
